# Effect of nitrogen supply method on root growth and grain yield of maize under alternate partial root-zone irrigation

**DOI:** 10.1038/s41598-019-44759-2

**Published:** 2019-06-03

**Authors:** Dongliang Qi, Tiantian Hu, Xue Song, Meiling Zhang

**Affiliations:** 1 Engineering Research Center of Ecology and Agriculture Use of Wetland, Ministry of Education, Yangtze University, Jingzhou, Hubei 434025 China; 2 Key Laboratory of Agricultural Soil and Water Engineering in Arid and Semiarid Areas of Ministry of Education, Northwest A&F University, Yangling, Shaanxi 712100 China; 3 Inner Mongolian Autonomous Regional Survey and Design Institute of Water Conservancy and Hydroelectricity, Hohhot, Inner Mongolia 010020 China

**Keywords:** Abiotic, Plant development, Plant development, Abiotic, Plant development

## Abstract

A field experiment was carried out to investigate effect of nitrogen (N) supply method on root growth and its correlation with the above-ground parts in maize (*Zea mays* L.) under alternate partial root-zone irrigation (APRI) at Wuwei, northwest China in 2012 and 2014. The treatments included alternate N supply, conventional N supply and fixed N supply under APRI (designated AN, CN and FN, respectively), with an additional CN fertilizer treatment coupled with conventional irrigation (CK). Ridges were built in a west-east direction. Root weight density (RWD) in the 0–100 cm soil layer and shoot biomass at the V_6_, V_12_, VT, R_2_ and R_6_ stages, and grain yield and yield components at the R_6_ were determined. Results showed that RWD around the plant (i.e. under the plant, south and north of the plant) in the 0–40 cm soil layer varied among different treatments at the VT, R_2_ and R_6_ stages. The RWD north and south the plant were comparable during maize growth stages for AN, CN and CK, while FN significantly decreased the RWD of its no N supply side at the three stages and markedly decreased the RWD of its N supply side at the VT. AN and CN significantly increased the RWD, shoot biomass at the three stages, and grain yield compared with FN and CK. Grain yield was positively correlated with RWD in the 0–40 cm soil layer at the three stages. These results suggested that AN and CN produced a relatively uniform distribution of roots and a greater root biomass, which contributed to the enhanced shoot biomass and grain yield of maize under APRI.

## Introduction

Plant roots systems are involved in acquisition of nutrients and water, synthesis of hormones, organic and amino acids, and anchorage of plants^1,2^. Crop growth and yield formation is significantly affected by roots^3,4^. Substantial research and practices have been undertaken to regulate root growth with the purpose of increasing grain yields^5,6^. Improving the ability of root absorption and its interception of nutrients and water is favored for enhancing crop yield and the efficiency of nutrient and water use, and reducing groundwater pollution^7,8^. In addition, root morphology is closely related with the growth and development of above-ground biomass^4,9^. Therefore, roots and their role in regulating absorption of nutrients and water has roused attention^4,10^.

It has been suggested that the spatial and temporal distribution of roots in soil is determined by soil water content, available nutrient in the soil and crop growth duration^11–13^. Irrigation methods affect distribution and dynamics of soil water content, which strongly influences the growth and development of roots^14^. For example, surface drip irrigation resulted in higher root length density in the 0–50 cm soil layer compared with border irrigation and sprinkler irrigation due to relatively more suitable soil water content^15^. Moreover, a localized nitrate or water supply could stimulate root growth in the nitrate or water supplied zone^16,17^. However, there is limited information on combined effects of localized nutrient and water supply on crop root systems^18,19^.

Alternate partial root-zone irrigation (APRI) is a new strategy of deficit irrigation, which is considered to be a water-saving irrigation technique and has been widely practiced worldwide^20–23^. In APRI, half of the root zone is irrigated while the other half is left dry, followed by irrigation of the previously dry root zone and drying of the irrigated half^20^. Under APRI, the situation is always that half of the root-zone is wet due to irrigation but the other half is left dry. However, usually nitrogen (N) supply is uniformly applied either as basal application or topdressing under APRI. Based on the fact that the interaction and complementary activities of nutrients and water play an important role in agricultural production^24^, it is necessary to explore an appropriate N supply method to improve the use efficiency of both N and water under APRI.

The Hexi Corridor area is one of the most important food production areas in China, where crop production is depends heavily on irrigation due to infrequent precipitation. In this area, Kang *et al*.^20^ studied the effects of varying irrigation methods on maize production, and found that APRI could maintain high grain yield with up to 50% reduction in irrigation water compared to conventional furrow irrigation. Moreover, when high efficiency water-saving irrigation technology was adopted, the fertilization method under original long-term irrigation conditions led to a large increase in volatilization of N fertilizer, which was not conducive to the sustainable development of agriculture^25^. However, to the best of our knowledge, the effect of different N supply methods on maize production, especially for spatial and temporal distribution of roots and its relationship with above-ground biomass in maize under APRI has yet to been addressed.

The objectives of this study were to (1) investigate root growth and distribution, and biomass accumulation at the V_6_, V_12_, VT, R_2_ and R_6_ stages, grain yield and yield components of maize in response to conventional N supply, alternate N supply and fixed N supply method, and (2) analyze the relationship between the root growth and grain yield or yield components under APRI. Conventional N supply is the N fertilizer application pattern frequently used by farmers, while alternate N supply and fixed N supply are two important approaches of localized N fertilization^18^. Therefore, the effects of the three N supply methods were tested in this study. The hypothesis is that an appropriate N supply method is beneficial to root growth and distribution, and consequently contributes to an increase in biomass accumulation and grain yield under APRI.

## Results

### Root weight density

Root weight density (RWD) is one of the most important parameters used for evaluation of roots. As shown in Table 1, RWD of maize in the 0–100 cm soil layer increased rapidly after the V_6_ stage and reached the maximum at the R_2_ stage, followed by a sharp decline until the R_6_ stage. At the R_2_ stage, the maximum RWD values among different treatments ranged from 12.57 to 13.56 g m^−3^ during 2012 growing season and 12.10 to 12.38 g m^−3^ during the 2014 growing season. There was no significant difference on RWD among different treatments at the V_6_ and V_12_ stages. However, compared to CK, AN and CN treatments significantly increased RWD at the VT, R_2_ and R_6_ stages in 2012 and 2014. RWD between FN and CK was comparable in 2012. Specially, RWD on average across these five growth stages for CN and AN treatments increased by 5.2% and 6.2%, respectively, in 2012 and by 7.6% and 7.0%, respectively, in 2014 compared with that of CK (Table 1).

**Table 1:** Effect of nitrogen supply method on root weight density (g m^−3^) in the 0–100 cm soil profile.

Year	Treatment	V_6_	V_12_	VT	R_2_	R_6_	Average
2012	CK	0.67a	7.22a	9.17b	12.63b	4.17b	6.77b
	CN	0.67a	7.21a	9.68a	13.47a	4.58a	7.12a
	AN	0.67a	7.37a	9.74a	13.56a	4.61a	7.19a
	FN	0.67a	7.24a	9.14b	12.57b	4.15b	6.75b
2014	CK	0.71a	7.43a	9.03b	12.10b	4.16b	6.69b
	CN	0.72a	7.50a	9.87a	13.21a	4.71a	7.20a
	AN	0.71a	7.41a	9.74a	13.28a	4.67a	7.16a

Values followed by different letters within each column are significantly different at the probability level of 0.05. Values are means (n = 3) of root weight density from north, south and under the plant in five layers of 0–100 cm soil profile.

### Horizontal root distribution

**Figure 1:**
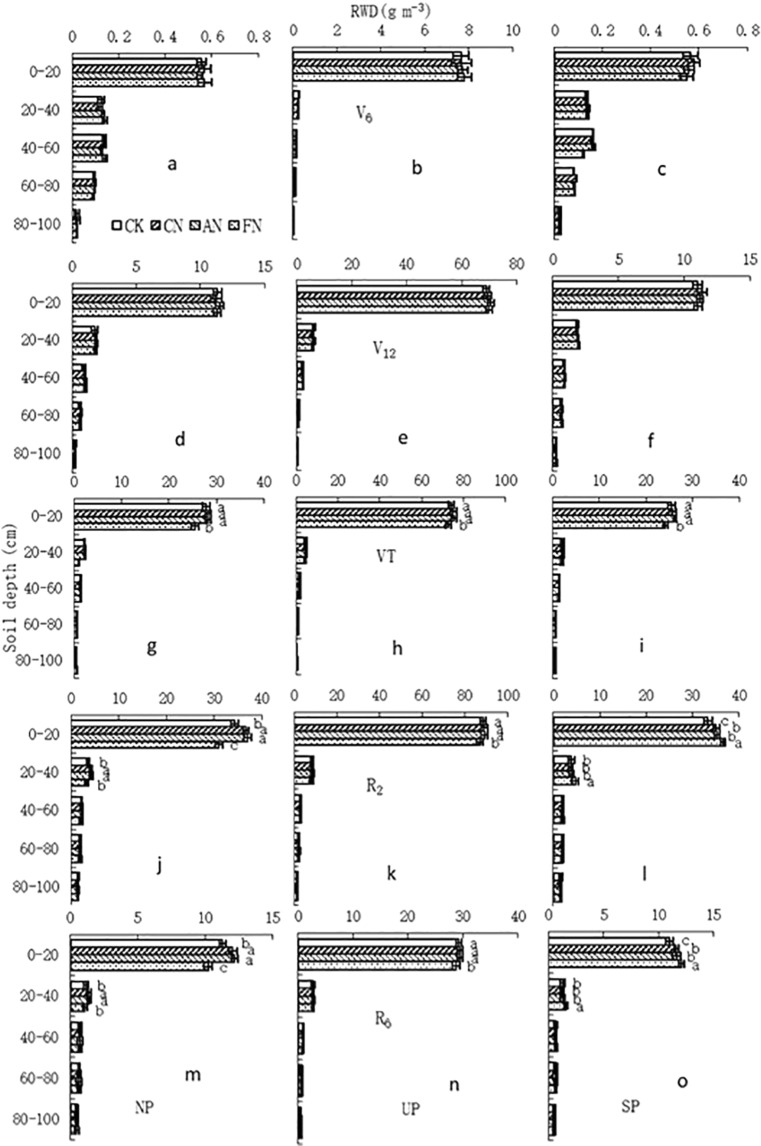
Effect of nitrogen supply method on root distribution of maize during the 2012 growing season. Note: RWD, root weight density; NP, north of the plant; UP, under the plant; SP, south of the plant. Values followed by different letters within each soil layer and sampling position are significantly different at the probability level of 0.05.

**Figure 2:**
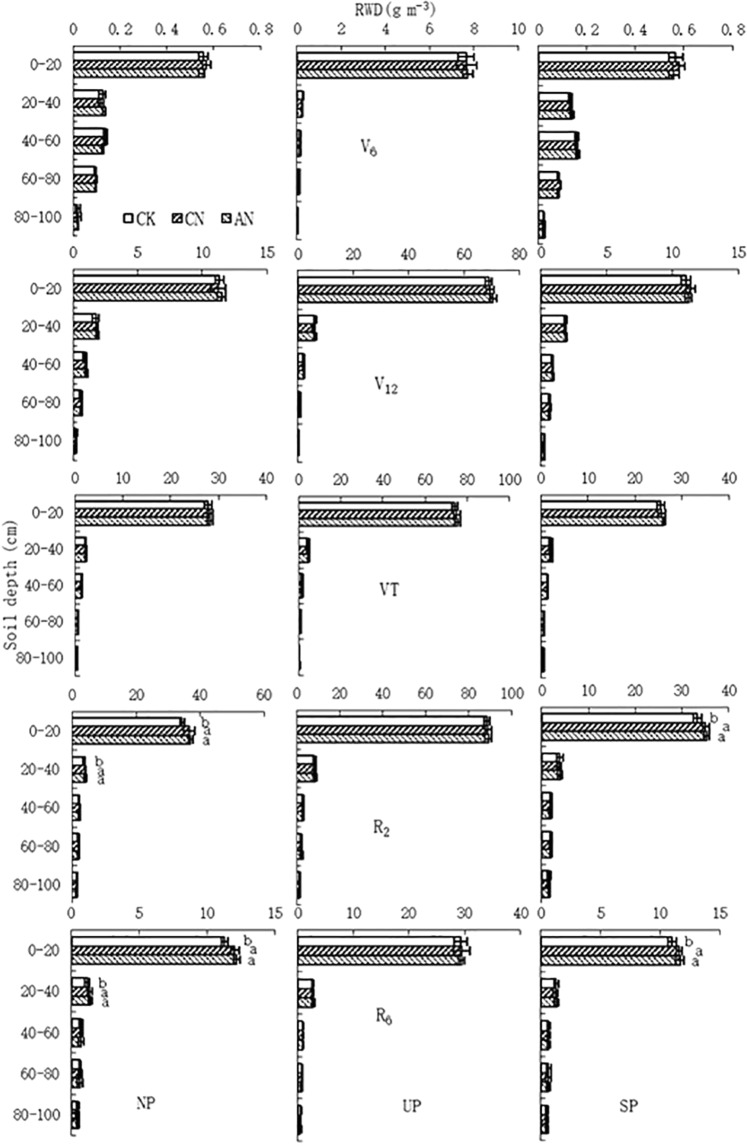
Effect of nitrogen supply method on root distribution of maize during the 2014 growing season. Note: RWD, root weight density; NP, north of the plant; UP, under the plant; SP, south of the plant. Values followed by different letters within each soil layer and sampling position are significantly different at the probability level of 0.05.

As shown in Figs 1 and 2, RWD in all treatments declined with soil depth, and significant differences were observed between soil layers. The root system was mainly distributed in the 0–20 cm soil layer, in which the RWD accounted for 83.5% and 82.4% of the sum of RWD in 0–100 cm soil layer (on average across these five growth stages) in 2012 and 2014, respectively. Moreover, RWD under the plant was significantly higher than RWD under north or south of the plant for all treatments.

Effect of nitrogen supply method on horizontal root distribution varied with soil depth and maize growth stages (Figs 1 and 2). In 2012, at the V_6_ and V_12_ stages, RWD at all three sampling locations (under the plant, to the north and to the south of the plant) were comparable among treatments for all five soil layers (Fig. 1a–f). However, at the VT stage, FN significantly decreased RWD in the 0–20 cm soil layer for all three positions compared to the other treatments (Fig. 1g–i). At the R_2_ and R_6_ stages, for samples taken from north of the plant, compared with CK, AN and CN significantly increased RWD in the 0–40 cm soil layer but FN significantly decreased RWD in the 0–20 cm soil layer (Fig. 1j,m). Under the plant, FN treatment significantly decreased RWD in the 0–20 cm layer soil layer compared with the other treatments (Fig. 1k,n). South of the plant, FN significantly increased RWD in the 0–40 cm soil layer compared with AN, CN and CK. And, AN and CN significantly increased RWD in the 0–20 cm soil layer compared to CK (Fig. 1l,o). RWD between AN and CN was comparable at all sampling times and sampling depths (Fig. 1). It is interesting that RWD in the 0–20 cm soil layer north and south of the plant was comparable at the V_6_, V_12_ and VT stages, while the RWD south of the plant was significant higher than the RWD north of the plant at the R_2_ and R_6_ stages for FN (Fig. 1g,i,j,l,m,o). In 2014, the root distribution by soil layer and growth stages for CK, AN and CN (Fig. 2) was very similar to the results obtained 2012 (Fig. 1).

### Shoot biomass

As shown in Table 2, shoot biomass at the V_6_ and V_12_ stages had no significant differences among the treatments. However, compared to CK, AN and CN significantly increased shoot biomass at the VT, R_2_ and R_6_ stages and FN treatment did not affect shoot biomass at these stages in 2012. A very similar observation was made on shoot biomass at the VT, R_2_ and R_6_ stages for CK, AN and CN treatments in 2014. And, shoot biomass at the VT, R_2_ and R_6_ stages for all treatments in 2012 were significantly higher than those in 2014.

**Table 2:** Effect of nitrogen supply method on shoot biomass (g plant^−1^) at different growth stages.

Year	Treatment	V_6_	V_12_	VT	R_2_	R_6_
2012	CK	14.5a	92.6a	145.6b	224.6b	281.4b
	CN	14.8a	93.1a	150.4a	231.5a	295.8a
	AN	14.2a	94.1a	151.2a	232.4a	294.6a
	FN	14.1a	92.9a	146.7b	222.9b	281.8b
2014	CK	15.4a	94.2a	141.2c	198.5d	237.6d
	CN	14.8a	93.2a	146.3b	204.2c	251.0c
	AN	14.6a	92.7a	147.0b	205.2c	253.0c

Values followed by different letters within each column are significantly different at the probability level of 0.05.

### Grain yield and yield components

As shown in Table 3, grain yield, ears per plant and 1000-kernel weight in all treatments in 2012 were significantly higher than those in 2014. CN and AN significantly increased grain yield while FN significantly decreased grain yield compared to CK. CN and AN significantly increased ears per plant and 1000-kernel weight compared to CK. The differences in ears per plant and 1000-kernel weight were not significantly different between CK and FN in 2012. A very similar observation was made on grain yield, ears per plant and 1000-kernel weight for CK, AN and CN treatments in 2014. However, kernels per cob were not significantly different among treatments in 2012 and 2014.

**Table 3:** Effect of nitrogen supply method on grain yield and yield components.

Year	Treatment	Grain yield (kg ha^−1^)	Ears per plant	Kernels per cob	1000-kernel weight (g)
2012	CK	7580b	1.37b	315.6a	293.4b
	CN	8415a	1.42a	321.5a	303.8a
	AN	8189a	1.40a	320.4a	304.5a
	FN	7228c	1.34b	311.4a	293.7b
2014	CK	5119e	1.06d	315.8a	277.5c
	CN	6774d	1.22c	307.5a	289.6b
	AN	6307d	1.21c	310.4a	285.4b

Values followed by different letters within each column are significantly different at the probability level of 0.05.

### Correlation between crop yield and root mass at five growth stages

The correlation coefficients between grain yield and yield components and RWD in different soil layers at five growth stages in 2012 and 2014 is shown in Table 4. Results shown that grain yield and ears per plant were significantly positively correlated with RWD in the 0–20 cm soil layer at the VT, R_2_ and R_6_ stages, and RWD in the 20–40 cm soil layer at the R_2_ stage. Kernels per cob were significantly positively correlated with RWD in the 0–20 cm soil layer at the R_2_ stage. 1000-kernel weight was significantly positively correlated with RWD in 0–40 cm soil layer at the R_2_ stage, and RWD in 0–20 cm soil layer at the R_6_ stage. No significant correlation was observed between yield or yield components and RWD at any soil depth at the V_6_ and V_12_ stages.

**Table 4:** Correlation analysis between grain yield, ears per plant, kernels per cob and 1000-kernel weight and root weight density by soil depth at five growth stages from data collected in 2012 and 2014.

Growth stage	Soil layer (cm)	Grain yield	Ears per plant	Kernels per cob	1000-kernel weight
V_6_	0–20	0.343	0.313	0.210	0.313
	20–40	0.215	0.215	0.310	0.254
	40–60	0.427	0.211	0.224	0.341
	60–80	0.314	0.180	0.123	0.467
	80–100	−0.217	0.124	0.217	0.313
V_12_	0–20	0.551	0.342	0.435	0.412
	20–40	0.451	0.427	0.324	0.356
	40–60	0.358	0.456	0.417	0.487
	60–80	−0.214	0.331	0.263	0.561
	80–100	−0.158	0.213	0.179	0.412
VT	0–20	0.755*	0.763*	0.434	0.414
	20–40	0.568	0.568	0.531	0.523
	40–60	0.462	0.631	0.613	0.557
	60–80	−0.421	0.527	0.542	0.345
	80–100	−0.432	0.418	0.315	0.627
R_2_	0–20	0.876**	0.754*	0.760*	0.883**
	20–40	0.762*	0.761*	0.614	0.759*
	40–60	0.637	0.537	0.531	0.458
	60–80	−0.248	0.424	0.425	0.561
	80–100	−0.131	0.218	0.133	0.324
R_6_	0–20	0.758*	0.759*	0.665	0.757*
	20–40	0.643	0.587	0.569	0.424
	40–60	0.587	0.435	0.458	0.536
	60–80	0.454	0.331	0.357	0.624
	80–100	−0.214	0.267	0.325	0.414

*P < 0.05; **P < 0.01.

## Discussion

In the present study, CN and AN were superior to FN and CK in terms of root growth beginning at the VT stage (Table 1). This was in line with the findings of Qi *et al*. that both conventional and alternate N supply generated a higher root length density at the R_6_ stage^19^. CN increased the root growth compared with CK; this was attributing to the alternation of wet and dry compartments, which resulted in compensatory root growth in the re-watered compartment after previous exposure to soil drying^17^. Moreover, it has been proved that soil N concentration in the 0–40 cm soil layer south of plant (N supplied) under FN was almost twice that under CN^26^. However, in the present study, RWD in the 0–40 cm soil layer at the VT, R_2_ and R_6_ stages south of the plant (N supplied) were not enhanced while the RWD north of the plant (no N supplied) were significantly reduced (Fig. 1). These may come from root growth inhibited by high concentrations of N^24,27^ due to south of the plant under FN had experienced two continuous applications of N fertilizer before the VT stage. In AN, N fertilizer was alternately applied to two adjacent furrows, resulting in N distribution between north and south of the plant was relatively uniform at the VT stage^26^. It has been suggested that the roots directly exposed to a localized N supply are stimulated because they benefit most from the increased N supply, or, alternatively, that increased metabolic activity in those same roots leads to a growth-stimulating influx of carbohydrates and auxin^28^. Thus, a localized N supply had both positive and negative effects on the root growth, which depended on soil N distribution.

In the present study, RWD was not significantly different in the 0–20 cm soil layer north and south of the plant at V_6_, V_12_ and VT stages, while south of the plant had greater RWD compared to north of the plant at the R_2_ and R_6_ stages for FN (Fig. 1). This might be due to the distribution of maize roots which was mainly in the 0–20 cm soil layer (Figs 1 and 2) and the rapid root growth that occurs between the V_6_ and VT^29^. At the early growth stages soil kept a certain level of nutrients thanks to a period of fallow between last harvest and this planting^30^. At the same time, the N requirement of maize is relatively small between the V_6_ and V_12_^31^, resulting in maintained root growth on the north side of the plant (no N supplied) for FN (Fig. 1a,d). With the rapid increase of N requirement of maize beginning at the VT stage^32^, original soil nutrients north of plant would be nearly depleted. In addition, root growth is sensitive to soil nutrients: roots tend to proliferate in high nutrient concentration regions^14^, resulting in enhanced root growth south of the plant (N supplied side) while root growth on the north side (no N supplied side) was decreased between the R_2_ and R_6_ for FN. Therefore we suggest that fixed nitrogen supply is not useful to achieve a uniform distribution of roots in maize under APRI.

Prior to this study, little information was available on root growth and it relationships with shoot biomass, grain yield and yield components in plants grown under varying N supply methods coupled with APRI. Our results showed that root growth was closely associated with shoot growth, grain yield and yield components (Tables 1, 2 and 4). It is suggested that an interdependent relationship exists between roots and shoots: active shoots ensure a sufficient supply of carbohydrate to roots and maintain root function; root function can, in turn, improve shoot characteristics by supplying a sufficient amount of nutrients, water, and phytohormones to shoots^1,31^. Moreover, Passioura demonstrated that roots are a major sink for assimilates, requiring twice as much photosynthesis to produce dry matter compared to shoots^33^. Based on this, redundant root growth is considered to cause inefficient consumption of energy and is not useful to improve shoot biomass accumulation and grain^34^. However, in the present study, the greatest RWD, shoot biomass and grain yield were found in CN and AN and the smallest were found in FN and CK (Tables 1, 2 and 3); no ‘redundant root growth’ phenomenon was observed. This was consistent with the findings of Wang *et al*. who demonstrated that improved root growth is useful to high grain yield^4^. Wang *et al*. insisted that water and nutrient absorption is a function of temporal and spatial distribution of the root system^4^. In addition, the growth and development of above-ground biomass depends on the acquisition of soil nutrients and water^35^. An increase in root biomass favored the promotion of photosynthetic production in above-ground plant parts, which ultimately increased grain yield^36^. Thus, here we speculate that a larger root system contributes to more nitrogen and water uptake from soil, and consequently, to a higher shoot biomass and grain yield for AN and CN.

Previous investigations have suggested that crop grain yield is closely related to the growth and development of roots, and that greater root biomass is associated with greater shoot biomass, which contributes to higher grain yield^3^. In addition, there is a greater effect on the yield of the shallow root system^37^. In this study, correlation analysis showed that grain yield and yield components were closely correlated to RWD in the 0–40 cm soil layer at the VT, R_2_ and R_6_ stages (Table 4). This finding indicates that beginning at the VT stage, root development in the 0–40 cm soil layer was very beneficial to generating a high grain yield of maize. This was associated with roots involved in acquisition of nutrients and water, synthesis of plant hormones, organic and amino acids, and anchorage of plants^1,2^. Also, maize roots are mainly distributed in the 0–40 cm soil layer (Figs 1 and 2). Moreover, since the VT stage, growth metabolism, N uptake and water use of maize are very vigorous^38,39^ and its growth enters into a crucial period for determination of ears per plant, kernels per cob and 1000-kernel weight^40^. However, the negative correlations between grain yield and RWD in deep soil layers (60–100 cm) were observed at almost all measured growth stages although none of these correlations were statistically significant (Table 4). We speculate that because of the low N concentration in these soil depths^26^, the nutrient uptake by plant is limited while the photosynthate input required to grow those roots is not reduced^41^. This suggests that the plant does not receive a good “return on investment” for growing the roots. Indeed, the reason is not clear and needs to be further investigated.

It can’t be ignored here that shoot biomass and grain yield were all greater in 2012 than 2014 across all treatments. This might be the result of different weather conditions between years. Unexpected high temperatures during grain filling (late August in 2014) may have contributed to the decreased biomass and grain yield in 2014^42^. Nevertheless, the patterns of root growth and distribution at the five stages, biomass accumulation and grain yield among N supply treatments were consistent among years, suggesting that the results are robust among varying environmental condition.

## Methods

### Experimental site

A field study was conducted during the 2012 and 2014 growing seasons at Wuwei Experimental Station for Efficient Use of Crop Water, Ministry of Agriculture, northwest China (latitude 37°52′20″N, longitude 102°50′50″E, altitude 1581 m). The site is in a typical continental temperate climate zone with mean annual precipitation of 164.4 mm, mean annual evapotranspiration of 2000 mm. Mean annual sunshine duration is over 3000 h and mean annual temperature is 8.8 °C. The groundwater level is consistently 40 m below the soil surface. The average air temperature, precipitation, and sunshine hours during the maize growing season across the two study years measured at a weather station within the experimental site are shown in Table 5. In the 0–40 cm soil layer, organic matter is 15.90 g kg^−1^, total N is 0.85 g kg^−1^, available N is 60.43 mg kg^−1^, total phosphorus is 0.93 g kg^−1^, available phosphorus is 6.22 mg kg^−1^, and available potassium is 236.24 mg kg^−1^.

**Table 5:** Precipitation, sunshine hours, and mean temperature during the growing season of maize in 2012 and 2014 at the experimental site.

	April	May	June	July	August	September
**Precipitation** (**mm per month**)						
2012	13	14	11	41	40	11
2014	20	17	12	46	75	5
**Sunshine** (**h per month**)						
2012	229	239	269	300	291	226
2014	213	226	279	312	259	235
**Mean temperature** (**°C**)						
2012	8.0	13.5	17.1	21.2	20.9	21.1
2014	7.6	14.2	17.2	22.2	22.3	21.6

Temperatures are the monthly averages.

### Crop management

Furrow irrigation was adopted in the field experiment. A trapezoid fracture surface was established for furrows and ridges. Furrows were 30 cm in depth and 20 cm in width at the bottom. Ridges were 20 cm and 35 cm in width at the top and bottom, respectively. This resulted in a ridge spacing of 55 cm. All experimental ridges were built in a west-east direction. Phosphorus was applied as triple superphosphate (P_2_O_5_ 46%) at a rate of 45 kg P ha^−1^ one day before furrows were established. Ridges were then covered using plastic film. Each plot was 24 m^2^ (4 m × 6 m) in 2012 and 32 m^2^ (4 m × 8 m) in 2014. Seven ridges were established for each plot in each year. Grain maize, cultivar ‘Golden northwest No. 22′ (*Zea mays* L.), was sown in the ridges at a density of 73000 plants ha^−1^ on 19 and 20 April in 2012 and 2014, respectively. The crop was harvested on 20 and 22 September in 2012 and 2014, respectively. Aside from the cultivation of maize for this experiment, for the remainder of the 2012 and 2014, the experimental field was fallow. Disease, weeds, and pests were well controlled in each treatment.

### Experimental design

The experiment was comprised of four treatments with three replications. The treatments included alternate N supply, conventional N supply and fixed N supply under APRI (designated AN, CN and FN, respectively), with an additional conventional N supply coupled with conventional irrigation (CK). In alternate N supply, N fertilizer was alternately applied to one of the neighboring two furrows in consecutive fertilizer applications. In conventional N supply, N fertilizer was applied to all furrows. In fixed N supply, N fertilizer was fixed to one of every two furrows. In APRI, one of the two neighboring furrows was alternately irrigated during consecutive waterings. In conventional irrigation, all furrows were irrigated during consecutive waterings. Based on the results of 2012, FN was excluded due to the obviously reduced the root growth and grain yield at the R_6_ stage compared to CK; only CK, AN and CN treatments were included in 2014.

Twice as much N was applied to the fertilized furrow in AN and FN treatments as that to the furrows in CN treatment, so that the total amount of N applied was identical for all treatments. Urea (46%) was applied at a rate of 200 kg N ha^−1^ to the center of the furrows and incorporated to a depth of 5 cm in soil, which is the optimum N rate for maize production in the local area^43^. N fertilizer application included basal application (50%) and topdressing at the V12 (25%) and VT (25%). The corresponding dates were 18 April, 14 July and 1 August, respectively in 2012 and 19 April, 12 July and 1 August, respectively in 2014. The irrigation amount of APRI was identical to that of conventional furrow irrigation. Irrigation was applied immediately after planting and at the V_6_, V_12_, VT and R_2_ of maize (75 mm per time), respectively. The irrigation water was supplied by a pipe with a diameter of 55 mm, and the amount of water applied was measured with a water meter installed at the discharging end of the pipe. Irrigation after the V_6_ and N fertilizer topdressing was conducted on the same day. The position details of localized irrigation and N application are described in Table 6.

**Table 6:** Position of localized irrigation and nitrogen fertilization at the different growth stages of maize.

Item	Sowing	V_6_	V_12_	VT	R_2_
Alternate furrow irrigation	South furrow	North furrow	South furrow	North furrow	South furrow
Conventional furrow irrigation	Both furrows	Both furrows	Both furrows	Both furrows	Both furrows
Alternate nitrogen supply	South furrow	/	North furrow	South furrow	/
Conventional nitrogen supply	Both furrows	/	Both furrows	Both furrows	/
Fixed nitrogen supply	South furrow	/	South furrow	South furrow	/

Note: “/” represents no treatment.

### Data collection

Before root sampling, the above-ground plant parts were sampled and dried to a consistent weight. Soil samples for root measurements were taken from three plants in the middle of each plot at the V_6_, V_12_, VT, R_2_ and R_6_ stages. A hand-driven auger (7 cm diameter) was used for sampling. The samples were collected to a soil depth of 100 cm from three positions around one plant. The three positions were: (1) directly over the crown of the plant (under the plant), (2) south and (3) north side of the plant directly opposite the crown (south of the plant and north of the plant)^19^. For positions (2) and (3), sampling sites were positioned one quarter of a row from the plant row (approximately 14 cm). The core was sectioned into 20 cm depths. According to Benjamin and Nielsen^12^, the samples were placed in a plastic, sealable bags and the bags were placed in refrigerated storage until washing the next day. Roots were washed from soil cores and debris and dead roots were removed from the samples. Samples were then dried at 75 °C to a constant mass and weighed. Root weight density (RWD) refers to root dry weight per unit of soil volume (3.14 × 3.5^2^ cm^2^ × 20 cm = 769.30 cm^3^) for each sampling, which was determined by the following formula^4^:

1 $$RWD=M/V$$

where RWD is root weight density (g m^−3^); M is root dry weight (g); V is the soil volume (m^3^).

According to Pandey *et al*.^39^, five plants (1.25 m row length) from each plot were sampled to collect grain yield components (kernels per cob, 1000 kernel weight, ears per plant) at maturity. The middle 6 m length of two rows was harvested to estimate grain yield at maturity.

### Statistical analysis

Analysis of variance (ANOVA) was performed using the general linear model-univariate procedure and the mean values were compared by Duncan’s multiple range test at the 5% level from SPSS 17.0 software. Pearson correlations were used to analyze the relationships between the roots systems and above-ground parameters.
